# Applying the Optimized CO Rebreathing Method for Measuring Blood Volumes and Hemoglobin Mass in Heart Failure Patients

**DOI:** 10.3389/fphys.2018.01603

**Published:** 2018-11-12

**Authors:** Christoph Ahlgrim, Philipp Birkner, Florian Seiler, Sebastian Grundmann, Manfred W. Baumstark, Christoph Bode, Torben Pottgiesser

**Affiliations:** ^1^Center for Medicine, Institute for Exercise and Occupational Medicine, Medical Center – University of Freiburg, Freiburg, Germany; ^2^Faculty of Medicine, University of Freiburg, Freiburg, Germany; ^3^Department of Cardiology and Angiology I, Heart Center Freiburg University, Faculty of Medicine, University of Freiburg, Freiburg, Germany

**Keywords:** CO rebreathing, hemoglobin mass, plasma volume determination, red cell volume determination, blood volume determination, heart failure

## Abstract

**Introduction:** Determination of blood volume, red cell volume, and plasma volume contributes to the understanding of the pathophysiology in heart failure, especially concerning anemia and volume load. The optimized carbon monoxide (CO)-rebreathing method (oCORM) is used to determine these parameters and hemoglobin mass (Hbmass) in exercise physiology. The applicability of oCORM to determine the intravascular volumes and Hbmass in heart failure patients is currently undetermined because assumptions concerning CO kinetics with oCORM rely on healthy subjects with a normal ejection fraction. Therefore, the aim of the present study is to determine the applicability and the systematic error of oCORM arising from a reduced EF when oCORM is used for measurement of intravascular volumes and Hbmass in heart failure patients.

**Methods:** oCORM was performed in 21 patients with heart failure and a reduced ejection fraction (EF) of < 30% (EFsev) and 25 controls (CONT). CO kinetics in capillary blood was studied 3–15 min after commencement of CO rebreathing. Differences in CO kinetics between the groups were assessed using a generalized linear model. The systematic error for determination of Hbmass with oCORM arising from differences in CO kinetics was assessed using the Monte Carlo method.

**Results:** The CO kinetics was significantly different between EFsev and CONT. In both groups, exposure to CO led to a COHb increase to 6.0 ± 1.0% 3 min after CO rebreathing. There were no CO related side effects or any clinical symptoms. Monte Carlo simulation quantifies the systematic error for determination of Hbmass arising from an impaired ejection fraction to be −0.88%.

**Conclusion:** Our results indicate an impaired vascular mixing of CO when EF is severely reduced. When Hbmass is determined using the original oCORM protocol in heart failure patients with a reduced EF, the systematic underestimation of about 1% should be considered. However, the error arising from this impaired vascular mixing appears small and clinically negligible. Furthermore, application of oCORM was safe and not related to any side effects resulting from CO exposure. In conclusion, oCORM can be used for assessing intravascular volumes and Hbmass in patients with a reduced EF.

## Introduction

Total BV and its constituents total RCV and PV have already been studied in heart failure patients decades ago (Eisenberg and McCall, 1954). Recent work demonstrated the importance of quantification of these parameters for understanding the pathophysiological adaptation in patients with cardiac failure as follows (Miller and Mullan, 2014). New insights into pathophysiology were provided when RCV was studied to understand the response to erythropoietin in patients with heart failure and preserved EF (Mancini et al., 2003; Borovka et al., 2013). PV was studied to document the shift of fluid through the body compartments in patients with decompensated heart failure (Miller and Mullan, 2014). In addition, hemodilution seems common in heart failure patients and anemia associated with a poor prognosis when I^131^-tagged albumin was used to measure RCV and PV (Androne et al., 2004). RCV and PV were related to differentiate the origin of anemia in patients with preserved or low EF where an expanded PV and pseudoanaemia have been found and the body’s RCV was not reduced (Adlbrecht et al., 2008).

Carbon monoxide (CO) can be used as a tracer for the corpuscular compartment to determine Hbmass, and derivatively, RCV and the other vascular volumes yielding a comparatively low measurement error, similar to the gold standard of red blood cell-labeling with radioactive chromium (51Cr) for measurement of blood volumes (Gore et al., 2005). The main advantages of using CO compared to 51Cr are the absence of radioactivity and an inexpensive tracer substance.

Various experimental set-ups for determination of these vascular parameters from inhalation of CO have been proposed. The most recent approach, the so called optimized CO-rebreathing method (oCORM) (Schmidt and Prommer, 2005; Keiser et al., 2015), appears suitable for application in patients in a clinical environment (Ahlgrim et al., 2014), especially due to a short rebreathing period of CO which appears as a further advantage compared to 51Cr-labeling. The method consists of inhaling and rebreathing a bolus of CO through a spirometer for 2 min and an analysis of the increase of carboxyhemoglobin (COHb) content of capillary blood at about 7 min after inhalation of CO. The method has been used in various settings in exercise physiology with respect to hypoxic exposure (Saunders et al., 2010, 2013; Ryan et al., 2014), training (Heinicke et al., 2001; Keiser et al., 2015) as well as cardiac adaptations (Ahlgrim et al., 2009). It is not routinely applied in clinical medicine. However, the risk profile of the method was analyzed recently in a small group of 18 cardiac patients with coronary artery disease (Karlsen et al., 2016), which demonstrated its safety in this patient population. Furthermore, oCORM was well tolerated by other patient groups such as patients with polycythemia vera (Ahlgrim et al., 2014).

CO rebreathing methods estimate Hbmass correctly only when the COHb level has equilibrated through the vascular system, meaning that a complete mixing between arterial and venous blood has occurred (Chada and Bruce, 2012). Different vascular mixing times ranging from 3 to 12 min (depending on the CO rebreathing method) have been proposed (Chada and Bruce, 2012). For oCORM, a complete mixing was assumed initially at 7 min after commencing rebreathing (Schmidt and Prommer, 2005). Noteworthy, a subsequent study highlights that, even in healthy subjects, a longer mixing time of up to 10 min might occur (Garvican et al., 2010).

Cardiac output is known to influence the vascular mixing time (Bruce and Bruce, 2003; Chada and Bruce, 2012). Although left ventricular EF should not be considered an approximation of cardiac output and both are only moderately correlated at rest (Carlsson et al., 2012), EF is one important determinant of cardiac output. Therefore, an impaired EF of patients with chronic heart failure might lead to an increased vascular mixing time and hence to false readings of Hbmass when oCORM is used. It is therefore unknown whether the conventional oCORM and associated formulas can be applied to study vascular parameters in this specific group of patients with chronic heart failure. It is further unknown at which time point the capillary samples need to be taken to ensure most accurate results.

Methodological issues need to be evaluated in order to adequately interpret and assess the data in a pathophysiological context, especially when patients with chronic heart failure are investigated. Various questions in cardiac pathophysiology could be addressed using this relatively simple procedure in the future. Therefore, the purpose of this study is to test the applicability of oCORM and quantify its systematic error in two groups of patients stratified by their left ventricular systolic function.

## Materials and Methods

### Subjects

For this study, 105 subjects were recruited and participated in CO-rebreathing. Subjects were recruited from the outpatient clinic of the University Heart Center and the Institute of Exercise- and Occupational Medicine. All subjects gave their written informed consent for participation in this study. The study was designed in line with the latest revised form of the Declaration of Helsinki and approved by the University Freiburg ethics committee. The study is an ongoing trial registered in the German registry for clinical studies (DRKS-ID: DRKS00006078). Of the 105 subjects, 15 subjects were excluded from further analysis because the quality of the CO rebreathing procedure did not fulfill our strict quality control aiming at non-existence of any leakage of CO during the procedure. Furthermore, 12 subjects were excluded because of missing data, which would have affected statistical evaluation. The heart failure patients were stratified according to their EF (assessed by echocardiography): EFmod (EF = 30–54%, *n* = 16) and EFsev (EF < 30%, *n* = 21). Patients in EFmod were not analyzed further for this part of the study in order to increase effect size of a reduced EF for the statistical analysis. Patients with a lung disease were excluded from this study. COHb-kinetics of 16 heart-transplant recipients with normal EF were not investigated further.

In EFsev, eight subjects had an ICD, three patients had cardiac resynchronization therapy (CRT) systems, two subjects without defibrillation function (CRT-P), one subject with defibrillation function (CRT-D). One patient had a device for cardiac contractility modulation (CCM).

In CONT (*n* = 25) subjects were recruited through the outpatient department of the Institute of Exercise- and Occupational Medicine and presented without apparent signs or history of heart disease. These subjects were healthy non-athletes based on a self-reported activity level. For further subject characteristics and pharmacotherapy, see Table 1.

**Table 1:** Subject characteristics.

	CONT	EFsev
N	25	21
**Characteristics**		
Age	41 ± 14^∗^	56 ± 7
BMI	22.7 ± 2.6^∗^	27.6 ± 3.2
Sex	10 f/15 m	6 f/15 m
Ejection fraction	–	<30%
**Reason for HF**		
Ischemic CM		7
Myocarditis		2
Dilatative CM		9
Hypertensive CM		1
Valvular CM		2
***Implanted devices***		
*CRT(-D)*		3
*ICD*		9
***Pharmacotherapy***		
*Beta blocker use*		20
*AT-1 antagonist*		8
*ACE-inhibitor*		11
*Calcium channel blocker*		0
*Loop diuretic*		13
*Thiazide diuretic*		3
**Corpuscular parameters**		
Hct (%)	41.9 ± 3.7^∗^	44.2 ± 4.0
Hb (g/dL)	15.2 ± 1.4^∗^	16.2 ± 1.7
Hbmass (g/kg)	12.0 ± 2.5^∗^	10.6 ± 1.6
Hbmass (g)	867 ± 266	901 ± 240
CO uptake/2-min	92% ± 3%	92% ± 3%

*Data in “Characteristics” are mean ± SD. CM, cardiomyopathy; CRT(-D), cardiac resynchronization therapy (- with defibrillator); ICD, implantable cardioverter-defibrillator; AT-1, angiotensin 1; ACE, angiotensin converting enzyme. ^1^Calculated from capillary samples at 6 and 8 min, ^∗^different from EFsev.*

### Application of CO Rebreathing

oCORM was applied in all subjects and determines Hbmass through labeling hemoglobin (Hb) with inhaled CO, resulting in a temporarily increased COHb. The gas is inhaled as a bolus in a standardized way and rebreathed for 2 min after mixture with oxygen using a closed-circuit spirometer (SpiCO, Blood Tec, Germany). oCORM accounts for ambient pressure and CO loss due to respiration and binding to myoglobin. During the procedure, a portable gas analyzer (Draeger Pac 7000, Draeger, Germany) is used to monitor for CO leakage at the mouthpiece and at the spirometer. The amount of remaining CO in the rebreathing circuit is also quantified.

As it was proposed the standard procedure conducting oCORM, COHb was measured in capillary blood before as well as 6 and 8 min after administration of CO (Schmidt and Prommer, 2005). In order to study COHb kinetics, we took additional samples at 3, 10, 12 and 15 min after administration of CO.

Capillary sampling was performed from the earlobe. A hyperemising ointment (Finalgon, Boehringer Ingelheim, Germany) was used to standardize blood-sampling conditions. Each sample was drawn into a capillary tube with a volume of 55 μl (Radiometer Clinitubes 55 μl, Radiometer, Denmark) and immediately analyzed in a hemoximeter unit of a standard point-of-care blood gas analyzer (Radiometer ABL 700series, Radiometer, Denmark) for determination of COHb and hemoglobin concentration [Hb]. Two additional capillary samples were drawn into two further capillary tubes, each with a volume of maximal 50 μl (Hettich Standard, Hettich, Germany) and immediately used for determination of hematocrit (Hct) using a centrifuge (Hettich Mikro 20, Hettich, Germany).

Hbmass is calculated as follows (based on Schmidt and Prommer, 2005):

Hbmass = K^∗^MCO^∗^100^∗^(ΔCOHb%^∗^1.39) ^−1^
K = barometric pressure^∗^760^−1^^∗^[1 + 0.003661^∗^ temperature (°C)]
MCO = CO_administered_ – (CO_losttosystem/lung/exhalation_)
CO_administered_ = CO volume administered into the system
ΔCOHb% = difference between basal COHb and COHb in the blood samples after CO administration
1.39 = Hüfner’s number (ml CO^∗^Hb^−1^)

Intravascular volumes (RCV, PV, BV) can be calculated from the following formulas (Heinicke et al., 2001)

RCV = Hbmass/MCHC^∗^100
BV = RCV^∗^100/Hct^1^
PV = BV – RCV
MCHC = mean corpuscular hemoglobin concentration. For RCV calculation, Hct should be corrected to whole-body Hct by the factor 0.91 (Chaplin et al., 1953) (Hct^1^).

Not all values to calculate CO diffusion capacity are measured using oCORM. However, approximation of CO diffusion capacity is possible based on quantification of CO remaining in the spirometer system as well as CO that is lost until COHb sampling and a standardized CO rebreathing time of 2 min. From these parameters, it is possible to estimate the fraction of CO tracer that has passed the pulmonary alveoli, being proportional to CO diffusion capacity.

### Echocardiographic Assessment

Echocardiography of heart failure patients was performed using the echocardiograph Philips IE33 (Philips Healthcare, Hamburg, Germany) by several experienced physicians. The final report of each examination was written by either one of two experienced cardiologists. From the standard long axis parasternal view, short axis parasternal view as well as apical four and two chamber view, EF was determined either by the modified Simpson method or estimated by thorough visual analysis of regional wall motion and final visual classification. In the context of this study, we only report EF for group allocation.

### Statistics

#### Assessing the Impact of an Impaired Cardiac Output on COHb Kinetics

Data for this exploratory study was collected using SAS JMP 11.0. COHb kinetics after CO rebreathing was assessed by a general linear model (PROC GLM, SPSS 23.0, using polynomic pre-set) with time and group as fixed and subjects as random effects for contrasts between the respective time points. As described above, only complete datasets (that are: exams with valid readings of COHb available at all time points) were included in this analysis. In order to determine the magnitude of the differences as an indicator of effect size, COHb values at the respective time points were normalized for the value at 15 min after beginning of rebreathing assuming the slow, rather linear washout had been reached at this time. Differences of the normalized COHb values at the respective time points were also assessed with PROC GLM and parameter estimates for the mean group effect were compared using T statistics. An alpha level of 0.05 was chosen for all analyses.

#### Assessing the Impact of a Reduced Ejection Fraction on Hbmass

In order to assess the impact of a reduced EF on determination of Hbmass using oCORM, the mean effects at time points 6 and 8 min where obtained from a generalized linear model examining all data normalized for the respective value 15 min after rebreathing. The regression coefficients for the time^∗^group interaction and their standard errors were then used for a simulation based on the Monte Carlo method (Metropolis and Ulam, 1949) projecting the error on exemplary subjects with low, medium and high Hbmass.

The subjects used for the simulation were chosen deliberately from the subject pool. For simulation, all measured values were used for calculation of Hbmass with the established formula (Schmidt and Prommer, 2005). A systematic error for the observed COHb value at the respective time points (6 min, 8 min) was added as a factor for which a normal distribution was assumed. The average factor value was set as the regression coefficient of the time^∗^group interaction. The standard error of the regression coefficient was chosen for the standard deviation of the distribution. The impact of the systemic error arising from altered COHb kinetics was quantified by subtracting the simulated Hbmass value from the measured value (that is: without using an error term). 100.000 repetitions of the correction factors were simulated for each subject.

## Results

Due to missing data in at least one of the seven instants of COHb measurements, 12 subjects (11.4%) had to be excluded because of missing data, as stated in the Methods section.

### Safety of the Method

The rebreathing procedure was tolerated well. No cardiac adverse effects, such as chest pain, dizziness or shortness of breath, were reported by any of the studied subjects. Neither were any signs of CO toxicity reported such as headache or impaired vision. At time point “3 min” average COHb was 6.0 ± 1.0%, at time point “6 min” average COHb was 5.6 ± 1.0%. No COHb values above 10% were observed.

### COHb-Kinetics

**FIGURE 1:**
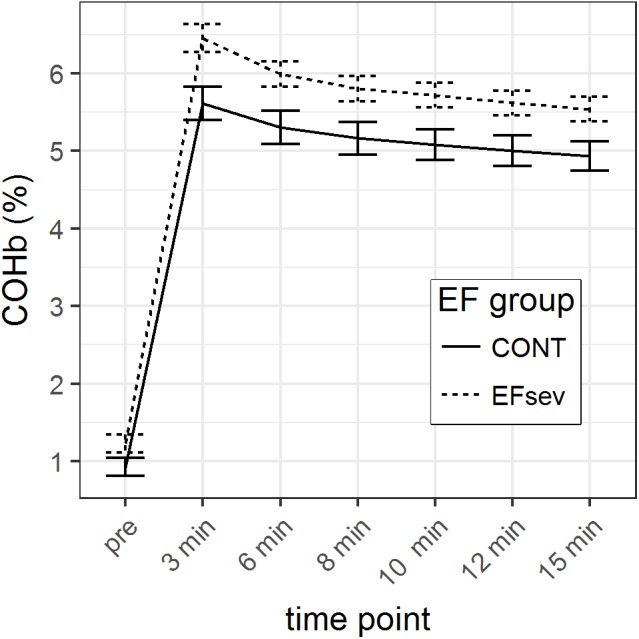
Course of carboxyhemoglobin (COHb) in capillary blood before and after rebreathing of carbon monoxide (CO) for 2 min using the optimized CO rebreathing method (oCORM). Data is displayed during a period of 15 min. Groups: CONT, control subjects; EFsev, subjects with EF < 30%.

In the linear model, we observed a significant interaction between COHb content at the respective time points and group (time^∗^group) to the third polynomial degree. Figure 1 depicts the mean COHb values illustrating an offset between the groups concerning COHb values. Thus, comparison of the differences of COHb content between groups was possible only using normalized data (normalized for the value at 15 min after beginning of rebreathing).

**FIGURE 2:**
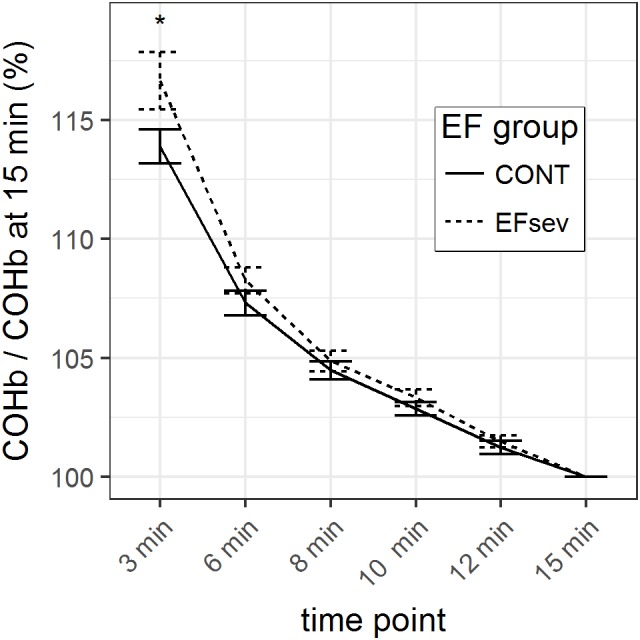
Course of carboxyhemoglobin (COHb) in capillary blood within 15 min after rebreathing of carbon monoxide (CO) using the optimized CO rebreathing method (oCORM), normalized to value at 15 min after rebreathing. ^∗^indicates statistical significant contrast between groups. Data provided as mean and standard error.

GLM parameter estimates of the normalized mean COHb values revealed a maximal difference between CONT and EFsev of 2.8% (*p* = 0.046) at 3 min after beginning of rebreathing which can solely be attributed to differences in COHb uptake/washout kinetics. At 6 min, this difference is 1.0% (*p* = 0.206), at 8 min the difference is 0.4% (*p* = 0.493), at 10 min the difference is 0.5% (*p* = 0.31), and at 12 min the difference is 0.3% (*p* = 0.484). The course of the normalized COHb values is depicted in Figure 2.

Considering the analysis of raw data and the analysis of the normalized data with the respective generalized linear models, it can be concluded that COHb values are converging after 3 min.

### Monte Carlo Simulation of the Impact of Reduced Ejection Fraction on Hbmass Determination Through oCORM

The generalized linear model provides the regression coefficient and standard error for the time^∗^group interaction at the respective time points 6 and 8 min after administration of CO. The regression coefficient/systematic error for EFsev group was + 1.0% [standard error (SE): 0.7%] at 6 min and + 0.4% (SE: 0.6%) at 8 min. Results of Monte Carlo simulation are displayed in Table 2.

**Table 2:** Monte Carlo simulation.

	Hbmass (g)	COHb pre (%)	COHb 6 min (%)	COHb 8 min (%)	CO circulation (ml)	K	Mean difference, range (g)	CV (range)
**Subject 1**	429	1.35	6.40	6.40	32.3	0.934	−4.1 (−8.3; 0.1)	−0.95% (−1.9%; 0.0%)
**Subject 2**	655	0.90	5.30	5.10	42.5	0.923	−5.7 (−11.9; 0,4)	−0.86% (−1.8%; 0.0%)
**Subject 3**	1234	0.85	5.40	5.30	82.9	0.931	−10.1 (−21.6; 1.1)	−0.82% (−1.7%; 0.0%)
								Average: −0.88%

*Hbmass values were calculated conventionally using the average of 6 and 8 min value; mean difference is (Hbmass_real_–Hbmass_sim_) in simulation, range indicates 5 and 95% percentile. K is the correction factor for barometric pressure and temperature according to the oCORM formula (Schmidt and Prommer, 2005).*

## Discussion

### Applicability of oCORM When Studying Subjects With Reduced EF

With this study, we demonstrate that oCORM appears applicable for measuring intravascular volumes and Hbmass even in patients with reduced EF. Key observation of the present study is that COHb kinetics after rebreathing a bolus of CO using oCORM is significantly different in subjects with a reduced EF compared to subjects with a normal EF. An equality of the slopes of COHb kinetics in capillary blood can be assumed 3 min after beginning of the CO rebreathing period. However, based on our calculations, it is now possible to quantify the effect of an impaired left ventricular systolic function on COHb kinetics and hence intravascular parameters conducting oCORM. For subjects with an EF below 30%, Hbmass values will be underestimated by 0.88% when using the conventional formula sampling COHb at 6 and 8 min (Schmidt and Prommer, 2005).

Our results prompt the following consequences when applying oCORM for studying intravascular volumes in subjects with a reduced EF:

(1) For highest accuracy, COHb samples could be drawn at 12 and 15 min after commencing CO rebreathing accounting for the different durations of vascular mixing time in order to reach the slow phase of COHb elimination kinetics. Whilst this procedure is remarkably different from the original experiment, this adaptation will minimize systematic error due to differences in mixing time between the studied subjects.
(2) When the original time points for sampling are used and an impaired EF is present or might be acquired in a longitudinal study setting, a systematic error of the method has to be accepted, which arises from a slight delay in CO distribution throughout the blood compartment when EF is severely reduced. However, from a clinical perspective, this systematic error of about 1% appears to be of a negligible magnitude based on our simulation. Depending on the individual Hbmass, this could lead to an additive systematic error of up to 1.9% based on our simulations (Table 2). For example, in subject 2, when a [Hb] of 13 g/dl is assumed, a difference of −5.7 g translates into an underestimation of BV by 44 ml.

The comparison with another method for RCV determination such as 51-chromium-labeling is appealing. The primary method parameter of oCORM is hemoglobin mass whereas the primary method parameter for 51Cr is BV, both with RCV as the final blood measure derived by equations. A meta-analysis showed that CO rebreathing yielded the lowest measurement error and is comparable with the gold standard 51Cr-labeling (Gore et al., 2005). The authors summarized that “Given the relative ease of handling CO compared with 51Cr, arising from the fact that [CO-rebreathing] is independent from biological variation in Hct, and the shorter biological half-life of CO, this review supports the routine use of CO rebreathing in clinical as well as research situations to monitor changes.” The rationale of our methodological paper is also supported by the recommendation of Gore et al that their “results also reinforce the importance of researchers estimating and reporting the error of measurement of the method in their hands to improve the analysis and interpretation of their data.”

Taking these considerations into account, using oCORM appears suitable to determine Hbmass in patient groups with heart failure.

### Safety and Failure Rate

Compared to the other few available studies of CO rebreathing in a clinical context (Ahlgrim et al., 2014; Karlsen et al., 2016), our study included a larger number of patients. With respect to safety of the procedure, we are able to contribute the safety data of 38 patients with an impaired systolic left ventricular function. The increase of COHb was similar to the values observed by Karlsen et al. (2016), who studied patients with coronary artery disease.

Besides impairing oxygen supply through blocking Hb, CO might theoretically exert detrimental effects in heart failure patients by binding to myoglobin. This could be relevant in heart failure patients, where arterial pO2 might be lower (Coburn et al., 1973). This issue was recently addressed by a simulation-based approach, suggesting that only a minor fraction of myocardial myoglobin is blocked by CO (Erupaka et al., 2010) during rebreathing. In our study, we did not observe any unwanted or harmful effects.

We stated a failure rate for oCORM of about 10% in a non-athletic, older population (Ahlgrim et al., 2014); a number that is in line with other spirometry assays (Ng’ang’a et al., 1992). In the current study, a higher rate of CO leakages resulting in test failure occurred (14.3%). In our opinion, this rather mirrors the rigorous quality control efforts as each occurrence of a leakage led to exclusion from analysis, which included several subjects with a leakage of less than 20 ppm. Most likely, such a small amount will not affect measurement results in a clinical setup. In addition, an optional dry run of the procedure (without CO) might lower the failure rate due to CO leakages.

Furthermore, 12 subjects were excluded from analysis because of incomplete capillary sampling. However, the method used for this study deviates from the “standard” method concerning the number of capillary samples. Whilst two capillary samples around the 7th min after beginning of rebreathing are intended by Schmidt and Prommer (2005), our study design yielded six capillary samples taken exactly at the specific time points in order not to confound the modeling of COHb kinetics. COHb values at 6 and 8 min after beginning of rebreathing exist for all subjects so that, in case the method was applied in clinical practice, no exclusions due to incomplete sampling would have occurred.

### Limitations

oCORM assumes similar CO diffusion for all subjects. From a clinical point of view, CO diffusion might be impaired in patients with severe heart failure, e.g., through development of post-capillary pulmonary hypertension (Simonneau et al., 2013). Noteworthy, oCORM is constructed very similar to experiments assessing CO diffusion capacity of the lung through rebreathing (Lewis et al., 1959). The ratio “CO uptake/2-min” is identical between the two groups (Table 1) which implies that in our study group results were not affected by differences in CO diffusion.

In the control group, echo was not performed. Although an impaired EF cannot be completely excluded, most likely, being free of any symptoms or medication, these subjects should yield a normal systolic cardiac function.

Moreover, only patients in a compensated state were studied. Therefore, it is unclear whether decompensated heart failure requires further adaptations of the sampling approach. The method seems unfeasible for decompensated patients on supplemental oxygen as CO administration might lead to a further deoxygenation. Hence, applicability and safety of oCORM in patients in a decompensated state, perhaps requiring supplemental oxygen, would need to be evaluated first.

## Conclusion

Our results indicate an impaired vascular mixing of CO when EF is severely reduced. When Hbmass is determined using the original oCORM protocol in heart failure patients with a reduced EF, the systematic underestimation of 0.88% should be considered. However, the error arising from this impaired vascular mixing appears small and clinically negligible. Furthermore, application of oCORM was safe and not related to any side effects resulting from CO exposure. In conclusion, oCORM can be used for assessing intravascular volumes and Hbmass in patients with a reduced EF.

## Author Contributions

CA and TP contributed to conception and design, analysis and interpretation of data, drafting, and revising the manuscript. PB, MB, and FS contributed to analysis and interpretation of data, and revising the manuscript. SG and CB contributed to conception and design, and revising the manuscript.

## Conflict of Interest Statement

The authors declare that the research was conducted in the absence of any commercial or financial relationships that could be construed as a potential conflict of interest.

## Abbreviations

BV: blood volume (ml)
CO: carbon monoxide
oCORM: optimized CO rebreathing method
COHb: carboxyhaemoglobin (%)
EF: ejection fraction (%)
Hbmass: total hemoglobin mass (g)
PV: plasma volume (ml)
RCV: red cell volume (ml).
